# Bubble-Wall Plot as a Dynamic Analytical Processing Visualization Tool for Developing Visual Warning Systems: a Case Study

**DOI:** 10.1371/journal.pone.0321077

**Published:** 2025-07-01

**Authors:** Robert M. X. Wu, Huan Zhang, Jie Liang, Niusha Shafiabady, Hai Yan (Helen) Lu, Ergun Gide, D. W. M. N. C. Dasanayake, Meena Jha, Shaoyang Duan

**Affiliations:** 1 School of Professional Practice and Leadership, Faculty of Engineering and Information Technology, University of Technology Sydney, Australia; 2 School of Geography, Shanxi Normal University, Taiyuan, China; 3 Visualisation Institute, School of Computer Science, University of Technology Sydney, Australia; 4 Discipline of IT, Australian Catholic University, North Sydney, Australia; 5 School of Computer Science, Faculty of Engineering and Information Technology, University of Technology Sydney, Australia; 6 Faculty of Engineering and Technology, Central Queensland University, Sydney, Australia; University of Hamburg: Universitat Hamburg, GERMANY

## Abstract

This paper proposes a dynamic analytical processing (DAP) visualization tool based on the Bubble-Wall Plot. It can be handily used to develop visual warning systems for visualizing the dynamic analytical processes of hazard data. Comparative analysis and case study methods are used in this research. Based on a literature review of Q1 publications since 2017, 23 types of data visualization approaches/tools are identified, including seven anomaly data visualization tools. This study presents three significant findings by comparing existing data visualization approaches. The primary finding is that no single visualization tool can fully satisfy industry requirements. This finding motivates academics to develop new DAP visualization tools. The second finding is that there are different views of Line Charts and various perspectives on Scatter Plots. The other one is that different researchers may perceive an existing data visualization tool differently, such as arguments between Scatter Plots and Line Charts and diverse opinions about Parallel Coordinate Plots and Scatter Plots. Users’ awareness rises when they choose data visualization tools that satisfy their requirements. By conducting a comparative analysis based on five categories (Style, Value, Change, Correlation, and Others) with 26 subcategories of metric features, results show that this new tool can effectively solve the limitations of existing visualization tools as it appears to have three remarkable characteristics: the simplest cartographic tool, the most straightforward visual result, and the most intuitive tool. Furthermore, this paper illustrates how the Bubble-Wall Plot can be effectively applied to develop a warning system for presenting dynamic analytical processes of hazard data in the coal mine. Lastly, this paper provides two recommendations, one implication, six research limitations, and eleven further study topics.

## 1. Introduction

Data visualization represents objects, situations, images, pictures, photographs, or sets of information in a diagram [1]. Literature shows that various terms are used in data visualization studies, including graphical visualizations, infographics, information graphics, informative graphs, and statistical graphics. Most recently, modern data visualizations have shaped the rapid development of information and communication technology (ICT) as an interdisciplinary scientific field that combines visualization, perceptual, and cognitive sciences [2–4]. They can analyze and interpret extensive and complex spatiotemporal data [5]. Furthermore, it may provide graphical representations of datasets to facilitate data analysis [6]. It will also facilitate insights and structures when used effectively [7].

Up-to-date literature shows that increased demands from industrial applications are impelling researchers and practitioners to seek more compelling data visualization tools for practical tasks [8–11]. They include seeking more practical visualization tools for less skilled or inexperienced users, presenting dynamic analytical processes of hazard data rather than the current static dataset focus, and discovering correlations between various dataset variables in warning systems. However, there are many challenges in creating more novel visualizations, which require moving beyond the idea of more and better information [1] and determining the best visualization tools [8,9]. Thus, seeking more effective visualization tools is required for given tasks.

This proposes a dynamic analytical processing (DAP) visualization tool based on Bubble-Wall Plot to address industry needs. This tool can be handily used to develop warning systems for visualizing the dynamic analytical processes of hazard data. Comparative analysis and case study methods are used in this research. A comparative analysis of existing data visualization approaches presents the gaps in data visualization tools for anomaly data visualization and also convinces the effectiveness of the new DAP visualization tool. To verify the state-of-the-art of the Bubble-Wall Plot and its novel, a case study is conducted using real-world datasets to demonstrate how this tool can be effectively applied to develop a warning system for presenting dynamic analytical processes of hazard data. The rest of the paper is organized as follows: Section 2 presents the demands and challenges in the field of data visualization with a focus on anomaly data visualization. Section 3 shows the related work. Section 4 describes A New Dynamic Analytical Processing (DAP) Visualization Tool in detail. Section 5 illustrates a case study of using the newly proposed tool in developing an early warning system in the coal mining industry. Section 6 presents recommendations, implications, and limitations. Section 7 draws conclusions and points out the focus of further research.

## 2. Demands and challenges

### 2.1 Demands

Research on data visualization indicates three increased demands for seeking novel and user-centered visualization tools. They include seeking more practical visualization tools for less skilled or inexperienced users, presenting dynamic analytical processes of hazard data rather than the current static dataset focus, and discovering correlations between various dataset variables.

The primary demand is that researchers and practitioners seek more practical visualization tools for less skilled or inexperienced users. Various visualization techniques have also supported human decision-making [12]. However, current data visualization tools are complex: users must first become experts in such fields [6]. Some techniques require end-users to know complex data outputs and have solid statistical backgrounds [13]. Other visualization tools require decision-makers to possess more complex data visualization knowledge [11]. However, inexperienced users can make incorrect decisions during the complex visualization process, which can have a negative effect on the final visualization outcomes [14].

The second demand is an increasing need to present dynamic analytical processes for hazard data rather than the current focus on static datasets. Data visualization tools should help users better understand processes [4] and ideally support the analytics processes of iterative inquiry into datasets to facilitate a better understanding of them [15]. They should also help researchers inspect the outputs of processes to discover and explore new patterns [4]. However, existing data visualization tools cannot describe the components of dynamic analytical processes among different hazard variables. Only a few studies have examined the analytical processes of visual discovery [3].

The third demand is to discover correlations between various dataset variables in warning systems. Data visualization tools should integrate data from the intersectionality of research models [16]. The existing tools do not have features related to dataset relationships for identifying correlations [11]. If data visualization tools could not identify the correlations between dataset variables, they often did not help the analysts [13].

### 2.2 Challenges

The literature has identified three challenges: challenging high-dimensional datasets, difficulties in identifying data patterns, and limited mapping data correlations.

- Challenging High-dimensional Datasets

The first challenge is that existing visualization tools face the challenge of visualizing high-dimensional datasets. Compelling visualization is vital for communicating with audiences unfamiliar with multi-dimensional and multi-scale information [2]. The necessity of combining multiple plots arises from the complexity of datasets with high-dimensional data; however, it is often difficult to convey all relevant information within a single chart type [17]. Current data visualization techniques are no longer effective due to the complexity and volume of data in industrial applications [18]. The increasing complexity of high-dimensional datasets has led to new challenges in the search for practical visualization tools [19]. With increasing hardware and soft resources, diverse and complex datasets are also growing [16].

- Difficulties in Identifying Data Patterns

Although data visualization has become a practical approach to understanding patterns [20], developing an effective user interface is challenging for identifying data patterns with a limited display. Discovering unexpected patterns in large datasets represents an internal cognitive activity that reveals unpredictable components inside otherwise hidden information between data and knowledge [2]. With adequate visualization methods, researchers can discover data patterns by employing their perceptual abilities [5] and leveraging human power [21,22]. The outputs should summarize the multivariate data into meaningful patterns for different large-volume datasets [2,23]. However, the nature of tasks and inherent goals have drastically changed pattern identification in the era of big data [11]. Furthermore, data visualization efforts have extended beyond a single graphic [24], which would challenge data visualization and statistical modeling within a single interface [9]. Offering too many allowable actions may increase interface complexity and distract users from making appropriate decisions [9].

- Limited Mapping Data Correlations

The existing visualization tools are limited to mapping data correlations between dataset variables. Studies have stated the need to place spatial and temporal dimensions into relations and visually correlate heterogeneous data from different dataset variables of application systems [5]. An effective visualization tool should be able to describe the correlations between variables in large datasets [23,25] and help users identify complex relationships [26]. The tool should aid in determining correlations and defining the cross-correlation patterns of different dataset variables [2,23] and even envision the relationships [27]. Many existing visualization tools do not have any relationship specifications [11].

## 3. Related works

This section reviews the literature and focuses on seven anomaly data visualization tools. It then reviews the metric features of these tools and points out different views on current research. Lastly, it highlights the findings and summarises the features of these tools.

### 3.1 Literature review

Researchers have reviewed data visualization studies before 2017, such as those by [9] and [10]. Thus, this study conducts a literature review of Q1 publications about data visualization between 2017 and 2024 and identifies 24 data visualization approaches (see Appendix 1). They include Bar Charts, Bubble Charts, Dimension Hierarchies, Geospatial Maps, Glyphs-based techniques, Heatmaps, Line Charts, Network Diagrams, Parallel Coordinate Plots, Pie Charts, Pixel-based techniques, Polar Coordinate Plots, Radar Charts, Radial visualizations, Sankey Charts, Scatter Plots, Stacked Graphs, Sunbursts, Table Lens, Tag Clouds, TreeMaps, Typographics, Violin Plots and Weather Maps.

### 3.2 Anomaly Data Visualization tools

*Anomalies* represent data points that differ from most others [8]. In this study, “outliers” refers to anomalies and outliers. They are used to identify abnormal values in a given dataset, e.g., statistical anomalies [28,29]. It is straightforward to verify that the observed value is inconsistent with other values usually present in the data [22].

Although the nature of data visualization tasks to identify outliers has changed drastically [30], most data visualization tools do not identify anomalies and outlines [31]. Among the approaches mentioned above, eight visualization tools can be used to identify outliers [8] They include Geospatial maps, Glyphs-based approaches, Line Charts, Parallel Coordinate Plots, Pixel-based techniques, Radial Visualizations, Scatter Plots, and TreeMaps.

Thus, this study focuses on seven anomaly data visualization tools used for general industrial applications rather than spatial information systems, excluding Geospatial maps.

### 3.3 Reviews of metric features of anomaly Data Visualization tools

Up-to-date research does not have standard anomaly specifications [11]. This paper collects ten critical metric features to characterize an anomaly data visualization tool from existing work [8,28,29,31]. They include: 1) *Retrieve Value* which is defined as the ability to retrieve the value of a particular visualization feature; 2) *Filter* which is defined as the ability to find data cases satisfying the specific conditions; 3) *Derived Value* which is defined as the ability to compute an aggregate numeric representation – such as average, median, and count – of a set of data cases; 4) *Find Extremum* which is defined as the ability to find an extreme value of an attribute over its Range within the data set; 5) *Determine Range* which is defined as the ability to identify the Range of their values; 6) *Distribution* which is defined as the ability to characterize the distribution of that attribute’s value over the dataset; 7) *Find Anomalies* which is defined as ability to identify any anomalies or statistical anomalies within a given dataset with respect to a given relationship or expectation; 8) *Find Clusters* which is defined as the ability to put visualization features of similar values into the same cluster and report the number of clusters; 9) *Correlation* which is defined as the ability to depict the relationships between the different data points; and 10) *Sort/order* which is defined as the ability to rank a set of given datasets according to a specific ordinal.

Fourteen functional features related to anomaly data visualization tools are also collected from business and industry viewpoints: *Low Dimensions, High Dimensions, Interaction, Layout Flexibility, Data Overview, Overplotted, Change Detection, Perceived Signal, Judgment, Performance Speed, Hierarchical Data Only, Data/Ink Ratio, Difficult in Ratios, and Details Limited*. They are discussed in the following subsection using the related tools.

The following subsection reviews the metric features of the seven anomaly data visualization tools.

- Glyphs-based Approaches

Glyphs-based approaches have multi-dimensions [32] and represent data points that can be positioned independently [8]. Behrisch, Blumenschein (8) discuss two significant advantages of glyphs. The first advantage of this layout is its flexibility. Another advantage is that glyphs can identify anomalies, clusters, and trends. The main limitation of this approach is that Glyphs-based approaches cannot carry any information about the datasets.

- Line Charts

A Line Chart uses the x and y axes to reference two directions in a coordinate system, which is widely used to visualize temporal data [8,33]. Line charts have several advantages [22]. A line chart is one of the best approaches to identifying correlations *because it has* the best accuracy, response time, and user preferences. Another significant advantage of Line Charts is that they provide better accuracy, response time, and user preferences when *determining Range*. The third advantage of Line Charts is that they have better accuracy and response time when *finding the Extremum.* The other benefits of the proposed tool include improved accuracy in *finding anomalies* and *filters* and better response time for *order*ing.

The main limitation is that selecting an appropriate aspect ratio of the height to width of a Line Chart is crucial. An inappropriate balance might lead to a wrong perception of judgments of value accuracy [8,34]. Another limitation is that Line Charts are surprising because of their low performance [22]. These include worse response time and user preference for *finding anomalies* and *filters*; worst user preference for distribution; worse accuracy and user preference for *order,* worse accuracy, worse response time, and worse user preference for *finding clusters* and *derived values;* worse user preference for *finding Extremum;* and worse *accuracy,* worse *response time,* and the worst user preference for *retrieving values*.

- Parallel Coordinate Plots

Parallel Coordinate Plots are widely used [30]. The first significant advantage of Parallel Coordinate Plots is that they provide a better overview of the data than other visualization techniques [31]. They are also better suited for displaying a good overview for users of higher-dimensional datasets [35,36]. The second significant advantage of the proposed tool is its ability to identify anomalies. Parallel Coordinate Plots can efficiently identify anomalies and support the observation of anomalies [31,37]. Through a parallel coordinates plot, every relationship between dataset variables can be plotted; any missing or incorrect data can be indicated visually [11]. The third advantage is its ability to identify or observe *correlations* [31,37,38]. The fourth advantage is its feature access to higher dimensions [8] and unlimited datasets [38]. Another advantage is that it can be used for several visualization tasks. Parallel Coordinate Plots can depict temporal data in *change detection* [37]. The sixth advantage is that Parallel Coordinate Plots may be better suited for obtaining insights into cluster shapes [37,39]. This approach yields higher accuracy, better response times, and better readability [35].

In addition, at least four challenges exist [8,37,40]: improper order leading to unfair practices, cluttered in higher dimensions, overplotting data to distort the patterns in higher dimensional datasets, and not supporting retrieving the value.

- Pixel-based Techniques

A Pixel-based technique creates a separate view for each dimension to map each pixel [8]. This technique encodes individual data values as pixels [41].

Behrisch, Blumenschein (8) discuss a few advantages. The first advantage of Pixel-based techniques is that they are well-used to identify *correlations, clusters, outliers, and trends: outliers* depicted with striking colors, clusters occurring in multiple dimensions, positive *correlations* identified with shared colors, negative correlations identified with consistently different colors, and trends depicted reoccurred of similar color. Another advantage of this pixel-based technique is that it is designed to display large datasets without aggregation and does not face overplotting problems. A significant limitation of the proposed method is that meaningful data patterns can only be identified if the *ordering* of dimensions is set appropriately.

- Radial Visualization

Radial visualizations can be used for each subindex’s dimensions [8,42]. The main advantage of radial visualization is that it can be helpful for static reports with no temporal aspect or focus on a single point in time, such as gaps or digital divides [42]. Another advantage is that radial visualization can identify *outliers, correlations, and trends* [8].

However, preference studies have indicated that Radars are the least helpful and popular [42]. Another limitation is that Radar is not recommended for interactive visualization [42]. Interactive visualizations may provide greater flexibility when directly interacting with datasets at multiple scales [4]. The third limitation is that Radial visualizations are highly dependent on the *ordering* of dimensions [8]: higher values in the two neighboring dimensions are closer to the circumference. The fourth limitation is that end users must understand the data details [42]. The fifth limitation is that this visualization has inherent limitations in terms of layouts and cannot show data details [43]. The clutter and overlapping features make radial visualizations difficult for end-users to identify values [42].

- Scatter Plots

The scatter plot shows the relationship between the two variables [8,14]. This approach has been widely discussed in the literature because of its significant advantages, such as [8,14,22,29,32,37,44,45].

The primary strength of the proposed tool is its ability to obtain a good overview of the data. The second significant advantage of the proposed tool is that scatter plots can help indicate *outliers*. The scatter plots had the best accuracy, better response time, and the best user preference for *finding anomalies*. The third advantage is that Scatter Plots are two-dimensional and can be extended to multi-dimensional data for data visualization. The fourth advantage of this tool is that scatter plots are often used to obtain an overview of the high-dimensional bivariate correlations. They may support correlation analyses between two variables. Scatter Plots have better accuracy and response time for *correlations.* The fifth advantage of the proposed method is that Scatter Plots can depict temporal data, facilitating visualization tasks in *change detection.* The Scatter Plots also demonstrate better accuracy, the best response time, and the best user preference for *determining Range.* The sixth advantage is that Scatter Plots have the best accuracy and response time for *finding Extremum.* The seventh advantage is that Scatter Plots have better accuracy and the best response time for *distribution.* The eighth advantage is that Scatter Plots have better accuracy for *order, derived value,* and *filtering.* Scatter Plots also have better response times for *ordering* and finding *clusters.*

Scatter Plots have four limitations and have been discussed in different studies, such as [14,22,45,46]. The first limitation is that they need to be more vital in presenting the data details. The second limitation is that Scatter Plots overlap with high superposition to visualize large datasets. The third limitation is that Scatter Plots cannot provide interactive functions. The fourth limitation is that Scatter Plots become more ineffective due to data growing in scale and complexity. Another limitation is that Scatter Plots have worse response times and user preferences for *derived* and *retrieved values.* Scatter Plots also have worse user preferences for *Correlation, distribution, order, filter, find cluster,* and *find Extremum.*

- TreeMaps

Treemaps can *identify outliers* [8]. Several studies have discussed its significant advantages, such as [8,47] and [32]. The primary advantage of the proposed model is the readability criterion. The eyes can quickly draw TreeMaps to outline and color rectangles for possible intervention. The second advantage of TreeMap is that it can efficiently use limited screen space. The third advantage of TreeMap is that it allows nodes to be resized smoothly to avoid data shuffle or occlusion. Another benefit is that TreeMaps may be suitable for micro/macro reading because they have a higher data-to-ink ratio. The data-to-ink ratio quantifies the efficiency and presents the data to the reader, similar to ink printed on paper [48].

TreeMaps have several limitations [8,49,50]: not be recommended for *identifying correlations,* not present zero value, and a negative value, only be applied to presenting hierarchical datasets, difficulty in presenting extreme aspect ratio of the height to width, encoding values using an area less accurate than a length.

### 3.3 Arguments on different views

Various viewpoints exist on the features of Line Charts and Scatter Plots, arguments between Scatter Plots and Line Charts, and diverse opinions about Parallel Coordinate Plots and Scatter Plots.

- Different Views of Line Charts

There is a different viewpoint on Line Charts in characterizing *distributions*. [22] find that Line Charts have better accuracy and better response time for distribution. However, [29] contend that Line Charts have worse accuracy and slower response times when characterizing *distributions.*

- Various Perspectives on Scatter Plots

There is a minor difference in the *distribution* of Scatter Plots. [22]mentioned that Scatter Plots had the best response time for *distribution*. [29] demonstrate that Scatter Plots have a better response time for *distribution*.

Other viewpoints may also be discussed. [29] comment that Scatter Plots have better response times for *Filtering*. However, [22] argue that Scatter Plots have worse response times. [29] believe that Scatter Plots have better perceptual accuracy for clusters. However, [22] argue that Scatter Plots have worse accuracy *for clusters.* [22] have highlighted that Scatter Plots have better retrieving value accuracy. However, [37] contend that they cannot demonstrate much strength to support *it.*

- Arguments Between Scatter Plots and Line Charts

A comparative analysis has been conducted between Scatter Plots and Line Charts [22]. Scatter Plots and Line Charts have better accuracy *for Filter*ing and better response times for *Order*ing and *Finding Extremum,* while Line Charts are much better. Scatter Plots are much better, while both have better accuracy for *Distribution and Determine Range*. Scatter Plots and Line Charts have worse response times and user preference for *filter* and *derived value*, worse accuracy and user preference for *find clusters*, and worse user preference for *order* and *Find Extremum, while* Line Charts have much worse.

- Diverse Opinions on Parallel Coordinate Plots and Scatter Plots

The results indicate different opinions on the performance of the Parallel Coordinate Plots and Scatter Plots. [44] have stated that researchers would like to generate output using parallel coordinate plots instead of Scatter Plots if the time for presenting data is limited. [35] believe that Parallel Coordinate Plots are better than Scatter Plots for *value retrieval tasks*. However, other studies have highlighted that scatter plots outperform or have an advantage over Parallel Coordinate Plots in the analysis of linear relationships [31], *judgment* accuracy [44], and for identifying data *cluster*s [35].

There are also different viewpoints on the features of low and high dimensions between parallel coordinate plots and scatter plots. [51] highlight that Parallel Coordinate Plots have advantages over Scatter Plots when they have low density and dimensions. However, [35] argue that Scatter Plots can achieve higher accuracy than Parallel Coordinate Plots for *low dimensions.* [51] believe that Scatter Plots outperform Parallel Coordinate Plots in higher dimensions and density datasets. However, [35] contend that Parallel Coordinate Plots can perform better than Scatter Plots in higher dimensions.

Research has also found that Scatter Plots have better response times than Parallel Coordinate Plots in lower dimensions (<8) and have better overall performance than Parallel Coordinate plots in depicting *correlations* [35,52].

### 3.4 Findings and summary of features

Based on the up-to-date literature and the aforementioned feature summary, this study finds that no single anomaly data visualization tool can cover all metric features to meet business and industry needs. A desirable anomaly data visualization tool should possess all these features. The second finding is that there are varying viewpoints on the metric features of the same visualization tool, such as different views of Line Charts and different perspectives on Scatter Plots. The third finding is that diverse opinions exist regarding the features of visualization tools. There are arguments between Scatter Plots and Line Charts. Diverse opinions have been expressed about Parallel Coordinate Plots and Scatter Plots. They need to be investigated in future research.

Appendix 2 summarizes the key findings for helping users better understand the 24 features and differences for each approach, including ten critical metric features (*Find Anomalies, Correlation, Distribution, order, Filter, Find Clusters, Derived Value, Find Extremum, Retrieve Value,* and *Determine Range)* and fourteen other functional features (*Low Dimensions, High Dimensions, Interaction, Layout Flexibility, Data Overview, Overplotted, Change Detection, Perceived Signal, Judgment, Performance Speed, Hierarchical Data Only, Data/Ink Ratio, Difficult in Ratios, and Details Limited*).

Thus, gaps exist between the features of existing visualization tools and the needs of contemporary businesses and industries. In practice, seeking more effective visualization tools for the given tasks is necessary.

## 4. A new Dynamic Analytical Processing (DAP) visualization tool

This section presents a new dynamic analytical process visualization tool based on the Bubble-Wall Plot. This method can mitigate the limitations of existing data visualization approaches for anomaly data visualization, as shown in Appendix 2.

### 4.1 Definition of DAP visualization tool

This tool consists of two parts. One is the Bubble-Wall Plot, and the other is a dynamic analytical process.

- Definition of a Bubble-Wall Plot

**Fig 1:**
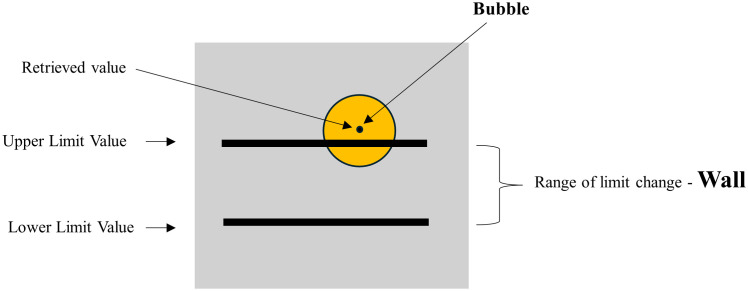
A Bubble-Wall Plot (extended from [54]).

The Bubble-Wall Plot is a one-dimensional plot comprising one bubble and one wall (Fig 1), which are described respectively below [53]:

The bubble with a circle shape represents the status of a target variable whose value has a safe range. The bubble can be defined by three attributes: (1) the position or center point, (2) the size or diameter, and (3) the color. The Wall represents the upper and lower boundaries of a normal value or the range of expected values of the target variable. It is represented by two parallel lines in this plot. One line represents the upper limit value (ULV) and the other represents the lower limit value (LLV).

- Determining attributes of the Bubble and Wall in a Bubble-Wall Plot

The status of the target variable determines the bubble’s color. By default, blue is for normal status, yellow is for anomaly, and red is for warning. The LLV and ULV can determine the wall. These two values are problem-specific; their default lower and upper limit values of the target variable.

The value of the target variable determines the bubble’s position for a given problem. Its radius is determined by the difference between the warning threshold value of the target variable and the upper boundary of the wall (ULV), i.e., r = TLV—ULV, where TLV represents the threshold limit value or the warning threshold value of the target variable and is usually known for a given problem domain. For example, in the coal mining industry, the warning threshold value of gas is 0.8 units.

- Dynamic analytical processing visualization tool

The DAP visualization tool is a Bubble-Wall Plot with a dynamic analytical process. A dynamic analytical process is described as achieving some objectives through monitoring a target variable for decision-making. The target variable value range needs to have a few segments. By default, the value range should have three segments: low anomaly, normal, and high anomaly. Each segment can be split into a few sections to indicate different safety statuses.

**Fig 2:**
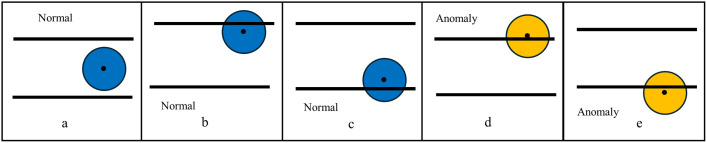
Five Statuses (a) Normal; (b) High Normal; (c) Low Normal; (d) High Anomaly; (e) Low Anomaly.

The tool can represent the status of the target variable, which is determined by the relative positions of the bubble, Wall’s ULV, and Wall’s LLV. By default, it can represent three statuses which correspond to the three default segments of target variable values, respectively, i.e., normal status (within the Wall) corresponds to the normal segment, high anomaly status (above the Wall) corresponds to the high anomaly segment and low anomaly status (below the Wall) corresponds to the low anomaly segment. If the target variable values have sections, the tool will offer corresponding statuses, respectively. For one instance, if a problem has a dynamic analytical process that has a target variable whose normal segment has three sections as normal, high normal, and low normal, the tool will offer a bubble-wall plot with five statuses as shown in Fig 2 [54], where (a) shows a normal status when the bubble position is near the middle of the Wall; (b) shows a high normal status when the bubble’s position is close but lower than the ULV line; (c) shows a low normal status when the bubble’s position is close but higher than the LLV line; (d) shows a high anomaly status when the bubble’s position is above the ULV line; and (e) shows a low anomaly status when the bubble’s position is below the LLV line.

**Fig 3:**
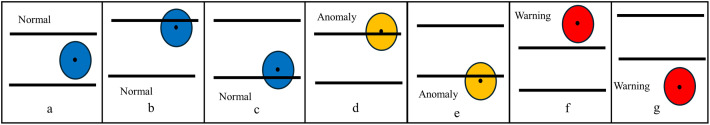
Seven Statuses **(a)** Normal; **(b)** High Normal; **(c)** Low Normal; **(d)** High Abnormal; **(e)** Low Fig Abnormal; **(f)** High Warning; **(g)** Low Warning.

For another instance, if a problem has a dynamic analytical process in which the target variable values have seven sections (3 for the normal segment and 2 for both high and low anomaly segments), the tool will offer a bubble-wall plot with seven statuses as shown in Fig 3, where (a)-(c) are the same as the ones in Fig 2, (d) shows a high abnormal status when the bubble’s position is above the ULV line but the bubble is partially in the Wall; (e) shows a low abnormal status when the bubble’s position is below the ULV line but the bubble is partially in the Wall; (f) shows a high warning status when the bubble’s position is completely above the Wall (The lowest point of the bubble is above the ULV line); and (g) shows a low warning status when the bubble’s position is entirely under the Wall (The highest point of the bubble is lower than the LLV line).

The Bubble-Wall Plots can be handily used for less trained or inexperienced users as the users (the safety-responsive management team) may only need to pay attention to the change in the bubble’s color: blue (normal status), yellow (anomaly), and red (warning) as follows:

-  If the Bubble value is lower than the ULV or higher than the LLV, the center point of the Bubble will be within the Wall, i.e., located between the lines of the ULV and LLV. They include the normal status (Fig 2a), the normal status with close but lower than the ULV (Fig 2b), and the normal status with close but higher than the LLV (Fig 2c). The bubble will be blue. The safety-responsive management team does not need to take action if the status is normal.
-  If the Bubble value exceeds the ULV or is lower than the LLV, the Bubble will be located above or below the ULV line. The bubble color synchronously changes from blue to yellow. Although this does not state any risks, the safety-responsive management team should carefully check the gas monitoring system to identify potential hazards. Examples of such anomalies include those exceeding the ULV (see Fig 2d) and an anomaly status lower than that of LLV (see Fig 2e).
-  If the Bubble value exceeds the ULV or is lower than the LLV, the Bubble will be located far above or below the ULV line. Examples of warning statuses that exceed the ULV (see Fig 2f) and a warning status that is significantly lower than that of the LLV (see Fig 2g). The bubble color synchronously changes from yellow to red. The safety-responsive management team must immediately take action in case of a warning status.

### 4.2 Procedure of using the DAP visualization tool

The DAP visualisation tool can be used for a monitoring system. The two compulsory conditions for using this tool are that the target variable be monitored and that the target variable has lower and upper limit values. If it is for an early warning system, warning high and low threshold values are also needed.

The procedure of using the DAP visualization tool consists of the following eight steps:

- Step 1. Define the problem statement
- Step 2. Collect data and perform data analysis
- Step 3. Define the target variable for the given problem and determine how to generate its values
- Step 4. Define the segments and/or sections of target variable values to determine the number of system statuses for the Bubble-Wall Plot
- Step 5. Calculate the upper and low limit values for the target variable to build the Wall, i.e., to determine the LLV and ULV values
- Step 6. Determine the TLV or the warning threshold values for the given problem. If there are no threshold values for the problem, use the default values as high TLV = ULV + (ULV-LLV)/2 and low TLV = LLV – (ULV-LLV)/2
- Step 7. Implement the Bubble-Wall Plotter for the target variable by setting up the Wall through the calculation of ULV and LLV and creating the bubble by determining its position by the target variable value. Its radius = high TLV—ULV or radius = LLV-low TLV, and the system status determines its color.
- Step 8. Based on the bubble’s color, determine the system status, such as normal or anomaly for a 3-status or 5-status system or normal, anomaly, or warning for a 7-status system.

### 4.3 Design considerations

Many visualization studies have focused on leveraging human vision to offload cognitive complexities in the past many years [15], such as [55–57]. However, a simple mantra for designing an effective and efficient visualization tool is first providing an overview and detail of the demand [5].

Therefore, this research uses two KIS strategies - keep it simple (KIS) and keep it the smallest dimension (KIS) – as a primary principle for designing the Buble-Wall Plot. The first KIS strategy is to keep it simple, comprising one Bubble and a Wall. Easy access to data visualization tools can increase user familiarity and improve user experience [11]. In other words, the simplest form is the most effective and efficient visualization approach for improving public research outcomes [8,16]. The more straightforward form is more intuitive to interpret [58]. The second KIS strategy is to use the smallest dimension possible. When using visualization idioms, one-dimensional lists should be used, and three- or more-dimensional visualizations should be avoided whenever possible [15]. The Bubble-Wall Plot is designed with the smallest spatial dimension – one dimension (Fig 1).

### 4.4 Design elements for the DAP Visualization Tool

[59] suggests using the seven essential design elements combined with visual semantics for linking data attributes, including position, form (shape), orientation, color hue, texture, color value (lightness or darkness), and size [60]. The Bubble-Wall plot can be well-depicted by four of Bertin’s design elements (color, shape, position, and orientation). Another three elements – texture, color value (lightness or darkness), and size – are not applied to designing this tool. They will be discussed in future research.

- Color Consideration

It is important to select colors for designing visualizations [61] because color schemes are applied to specific and recommended uses [62]. The color element is primarily considered.

The literature indicates that the three-light metaphor – green, yellow, and red – is frequently used for signals. The green light indicates a high likelihood of success or a normal status, whereas the yellow light indicates potential problems [12]. The red hue has a robust semantic association with warnings in most cultures. A common warning symbol indicates increased risks [58] and a high likelihood of failure or danger status [12]. However, some individuals could not identify the difference between green and red [63]. Psychophysics studies have shown that blue hue has been an attractive color to humans throughout history [63].

Therefore, this study uses the blue-yellow-red metaphor to design a Bubble-Wall Plot: a blue hue presenting the normal status, a yellow hue indicating the anomaly, and a red hue highlighting the warning status.

- Shape Design

The circle symbol (shape) represents systems with normal, anomalous, and warning statuses. The blue circle represents the normal states (see Fig 3a–3c). The yellow circle represents the anomaly status (see Fig 3d–3e). The red circle in Fig 1 indicates the warning status (see Fig 3f–3g).

- Position

The bubble location represents seven statuses. If the Bubble value is lower than the ULV or higher than the LLV, the Bubble will be located between the lines of the ULV and LLV. They include the normal (Fig 3a), being close but lower than the ULV (Fig 3b), and being close but higher than the LLV (Fig 3c). If the Bubble value exceeds the ULV or is lower than the LLV, the Bubble will be located above the ULV line or below the LLV line. These include anomalies that exceed the ULV (see Fig 3d) and anomaly status lower than that of LLV (see Fig 3e). If the Bubble value exceeds the ULV or is far lower than the LLV, the Bubble will be located above the ULV line or below the LLV line. They include warning statuses that exceed the ULV (see Fig 3f) and a warning status that is significantly lower than that of the LLV (see Fig 3g).

- Orientation Feature

The orientation feature is also well-considered in this novel DAP tool, including two directions: being close to the ULV or exceeding the UL and being close to the LLV or lower than the LLV. Three statuses are close to the ULV or exceeding the UL, including the normal status with close but not exceeding the ULV (see Fig 3b), anomaly status that exceeds the ULV (see Fig 3d), and a warning status that far exceeds the ULV (see Fig 3f). Three statuses are close to or lower than the LLV, including the normal status, which is close but not lower than the LLV (see Fig 3c), anomaly status with lower than the LLV (see Fig 3e) and a warning status that is significantly lower than that of the LLV (see Fig 3g).

### 4.5 Comparative analysis

A comparative analysis explains how this newly designed DAP tool appears novel compared to the above seven approaches by focusing on five categories (Style, Value, Change, Correlation, and Others), including 26 subcategories (see Appendix 3). Researchers and practitioners seeking a suitable data visualization approach can adopt these categories. The references in this section have been discussed or cited in section 3.3 (*Features of Anomaly Visualization Tools*) and section 3.4 (*Arguments on Different Views*).

- Style

The style features focus on seven subcategories: *Symbolization Style, One Dimension Type, Low Dimensions, High Dimensions, Ordering of Dimensions, Interaction,* and *layout flexibility.* These subcategories include the following:

- For *Symbolization Style*, the Bubble-Wall Plot addresses the first KISS strategy comprising one Bubble and two horizontal lines. It is the most straightforward style compared to the other seven approaches. Line Charts and Scatter Plots have a more straightforward symbolization style than Glyphs-based approaches, Parallel Coordinate Plots, Pixel-based techniques, Radial visualizations, and TreeMaps.
- For One-*Dimension Type*, the Bubble-Wall Plot addresses the second KISS strategy with a one-dimensional design—the smallest number of spatial dimensions. Line Charts are two-dimensional. Glyphs-based approaches, Parallel Coordinate Plots, Pixel-based techniques, Radial visualizations, and TreeMaps have multi-dimensions. Scatter Plots are two-dimensional and cannot be extended to higher dimensions for larger datasets.
- For *Low Dimensions (two dimensions)*, the dimension datasets could be transformed into one-dimensional and adopted in the Bubble-Wall Plot (see a demo of two dimensions in Chapter 5 Case Study). Parallel Coordinate Plots and Scatter Plots are suitable for low-dimensional visualization. However, there are different perspectives. One study believes that Parallel Coordinate Plots have advantages over Scatter Plots with low density and dimensions. Simultaneously, another studies argue that Scatter Plots achieve higher accuracy for lower dimensions than Parallel Coordinate Plots and have better response times than Parallel Coordinate Plots in lower dimensions (<8). Pixel-based techniques create a sub-window and color for each dataset in respective dimensions according to the value. Radial visualizations can be used for any dimension in which each subindex may have. No previous studies have considered this feature in Glyphs-based approaches, Line Charts, or TreeMaps.
- For *High Dimensions (three or more dimensions)*, the Bubble-Wall Plot is a one-dimensional tool. The higher-dimensional datasets needed to be transformed into one-dimensional datasets to fit the use of the Bubble-Wall Plot (see a demo of two dimensional datasets resformed into on-demention in Chapter 5 Case Study). Glyphs-based approaches can represent high-dimensional data. Both Parallel Coordinate Plots and Scatter Plots are suitable for high-dimensional visualizations. A previous study reports that scatter plots outperform parallel coordinate plots in *high dimensional* and density datasets. However, another study shows that Parallel Coordinate Plots perform better for higher dimensions than Scatter Plots. Pixel-based techniques can represent *clusters* and *outliers* in *higher dimensions*. Radial visualizations can be used for any dimension in which each subindex may have. TreeMaps allow the *display of high-dimensional data* to efficiently depict hierarchical data aspects. No previous studies have discussed this feature in Line Charts.
- For *Ordering of Dimensions*, the Bubble-Wall Plot uses a one-dimensional approach to order dimensions. Line Charts have worse accuracy, better response time, and worse user preference for *orders*. A Parallel Coordinates Plot depends on ordering the dimension that appreciates ordering, which could enhance Correlation and clustering and reveal unknown patterns. Pixel-based visualizations can only find meaningful data visualization patterns if the dimensions are ordered appropriately. Radial visualization is also highly dependent on the dimensional *ordering*. Scatter Plots have better accuracy and response times but worse user preferences for *orders*. No previous studies have investigated this feature in Glyphs-based approaches or in TreeMaps.
- For *Interaction*, the Bubble-Wall Plot is designed as an interactive approach. Interactive data visualization is a compact technology agnostic that captures human intent and interaction causality, including the full spectrum of human senses [9,64]. Radial visualizations are not recommended for interactivevisualization. Scatter Plots are limited to interactions that cannot provide interactive functions. No previous studies have discussed interactive processes using Glyphs-based approaches, Line Charts, Parallel Coordinate Plots, Pixel-based techniques, or TreeMaps.
- For *Layout Flexibility*, the Bubble-Wall Plot is the most straightforward tool with significant layout flexibility. Glyphs-based approaches are structurally flexible. A TreeMap is a good tool to visualize the design. No previous studies have examined this feature in Line Charts, Parallel Coordinate Plots, Pixel-based techniques, Radial visualizations, or Scatter Plots.

- Value

The features for the value category focus on seven subcategories, including *Find Anomalies, Find Extremum, Retrieve Value, Derived Value, Details Limited, Data Overview,* and *Overplotted*, including:

- For *Finding Anomalies*, the Bubble-Wall Plot can effectively identify anomalies. If the Bubble value exceeds the ULV or is lower than the LLV, it is an anomaly (Fig 2d–2e). Line Charts have better accuracy, worse response time, and worse user preference for *finding anomalies*. Parallel Coordinate Plots are efficient for *identifying outliers*. Scatter Plots have the best accuracy, better response times, and best user preference for *finding anomalies*. Glyphs, Pixel-based techniques, Radial visualizations, and TreepMaps can also identify *outliers*.
- For *Find Extremum,* the Bubble-Wall Plot is designed to *find the Extremum* effectively (see Fig 2d–2g). Glyphs-based approaches could not *find Extremum*. Line Charts have better accuracy, shorter response time, and worse user preference for *finding the Extremum*. A Parallel Coordinates Plot can present the highest and lowest values. Scatter Plots have significantly higher accuracy, shorter response time, and worse user preferences relative to *finding Extremum*. TreeMaps lacks a value message and cannot *find the Extremum*. No previous studies have discussed this feature in Pixel-based techniques or Radial visualizations.
- For *the Retrieve Value,* the Bubble-Wall Plot easily retrieves a particular value. Glyphs-based approaches could not *retrieve the value*. Line Charts have the worst user preferences, worse accuracy, and worse response times for *retrieving value*. The Parallel Coordinates Plot cannot support retrieving *value*. The overlapping and cluttering features make it challenging to identify and compare values in Radial visualizations. Scatter Plots are better with accuracy for retrieving *the value* but worse than Parallel Coordinate Plots for *value retrieval* tasks. However, another study contests that Scatter Plots can not demonstrate much strength in supporting *value retrieval*. Scatter Plots have worse response times and worse user preferences for *retrieving value.* The proposed TreeMap lacks a value message. No previous studies have discussed this feature in Pixel-based techniques.
- For *Derived Value*, the Bubble-Wall Plot is designed to compute the derived value (Chapter 5 Case Study). Glyphs-based approaches do not carry data values. Line Charts can present a *Derived Value,* but they have low performance. The overlapping and cluttering make Radial Visualizations are difficult for *derived values*. Scatter Plots have better accuracy but worse response times and user preference with the *derived value*. The proposed TreeMap lacks a value message. No previous studies have considered this feature in Parallel Coordinate Plots or Pixel-based techniques.
- For *Details Limited*, the Bubble-Wall Plot would provide details for data visualization. Radial visualization cannot show all data details. Scatter Plots are also weak in displaying pieces of data. No previous studies have investigated this feature in Glyphs-based approaches, Line Charts, Parallel Coordinate Plots, Pixel-based techniques, or TreeMaps.
- For *Data Overview*, the Bubble-Wall Plot may present a distinctly arranged view of the data. Parallel Coordinate Plots can present an *overview* of multidimensional data. A Scatter Plot can also provide a quick overview of the data. No previous studies have discussed this feature in Glyphs-based approaches, Line Charts, Pixel-based techniques, Radial visualizations, or TreeMaps.
- For *overplotting*, the Bubble-Wall Plot is a one-dimensional tool that does not suffer overplotting problems. Parallel Coordinate Plots face the challenges of *overplotted* lines with increasing datasets. A Pixel-based technique is designed to display large datasets without aggregation and does not face *overplotting* problems. Radar visualizations have a significant overlap. Scatter plots significantly overlap the high superposition presented to visualize large datasets. No previous studies have reviewed this feature in Glyphs-based approaches, Line Charts, or TreeMaps.

- Change

The features for the change category include four subcategories: *Determine Range, Change Detection, Perceived Signal,* and *Judgment.* These subcategories include the following:

- For *Determining Range*, the Bubble-Wall Plot can explicitly identify the range of values obtained by the ULV and LLV relative to data fluctuations in related datasets. Line Charts have better accuracy, response times, and user preference when *determining the range* of current visualization approaches. Scatter Plots have better accuracy, significantly higher response times, and user preference when *determining Range*. No previous studies have investigated this feature in Glyphs-based approaches, Parallel Coordinate Plots, Pixel-based techniques, Radial visualizations, or TreeMaps.
- For *Change Detection*, the Bubble-Wall Plot effectively detects changes through changes in color hue. If the bubble color changes from blue to yellow, the system is at an anomaly status. If the bubble color changes from yellow to red, the system receives a warning. Parallel Coordinate Plots have better accuracy and response time for sensitivity analysis tasks to change detection sub-datasets among current visualization approaches. Scatter Plots can also be used to facilitate change detection in visualization tasks*.* No previous studies have examined this feature in Glyphs-based approaches, Line Charts, Radial visualizations, Pixel-based techniques, and TreeMaps.
- For *Perceived Signal*, the Bubble-Wall Plot uses three pure color hues (blue, yellow, and red) to indicate three statuses (Normal, Anomaly, and Warning status). This tool uses a blue hue to state a normal status. Yellow hue is used to indicate anomaly status. The red hue highlights the warning status. Parallel Coordinate Plots generate outputs rather than Scatter Plots. No previous studies have reviewed this feature in Glyphs-based approaches, Line Charts, Pixel-based techniques, Radial visualizations, and TreeMaps.
- For *Judgment*, the Bubble-Wall Plot uses three hues (blue, yellow, and red) to assist decision-makers. Line Charts need to improve the accuracy of value *judgments.* Parallel Coordinate Plots have much lower *judgment* accuracy than Scatter Plots. A TreeMap needs to have more accurate *judgments*. No previous studies have reported this feature in Glyphs-based approaches, Pixel-based techniques, or Radial visualizations.

- Correlation

The features of the correlation category focus on the following four subcategories: *correlation, Finding Clusters, Filtering, and Characterizing Distribution,* including:

- For *Correlation*, the Bubble-Wall Plot is well-designed to present the relationships between the variables (Chapter 5 Case Study). Line Charts have the best accuracy, response time, and user preference for *Correlation* among current visualization approaches. Parallel Coordinate Plots can help observe *correlations* and quickly identify *correlation* patterns at a glance compared with other visualization tools. Pixel-based techniques and Radial visualizations can also be used for effective correlation. Scatter Plots have better accuracy and response times but have worse user preferences for *correlations*. No previous studies have discussed this feature in terms of Glyphs-based approaches, Radial visualizations, and TreeMaps.
- For *Find Clusters*, the Bubble-Wall Plot has not been designed. Among the current visualization approaches, Glyphs can be used to *identify clusters*. Line Charts can be used *to identify clusters; however, they exhibit low* performance. Line Charts have much worse accuracy and user preferences than Scatter Plots for *Find Clusters*. Parallel Coordinate Plots have better accuracy and response time performance than other visualization approaches to *clustering*. Parallel Coordinate Plots are better suited to obtain cluster shape insights. Pixel-based visualizations can also be used to *find clusters* in higher dimensions. Scatter Plots have better response times but worse user preferences for *finding clusters*. Although Scatter Plots have better perceptual accuracy for *clusters,* another study debates that Scatter Plots have worse accuracy for *finding clusters.* Scatter Plots have an advantage over parallel coordinate plots in identifying data *clusters*. TreeMap is one of the most relevant approaches for data *cluster* analysis. No previous studies have considered this feature in Radar visualizations.
- For *Filter*, the Bubble-Wall Plot does not satisfy this feature. Among the current visualization approaches, Line Charts have better accuracy, worse response time, and worse user preference for *filters*. Parallel Coordinate Plots are better suited for filtering high-dimensional source data. Scatter Plots provide better accuracy for the *filter*. However, there are different viewpoints on the response time to Scatter Plots for the *filter*: one study is believed to have a better response time; another research project argues that Scatter Plots have worse response times for the *filter*. Line Charts have much worse user preferences than Scatter Plots for *filters*. No previous studies have examined this feature in Glyphs-based approaches, Pixel-based techniques, Radial visualizations, and TreeMaps.
- For *Characterize Distribution,* the Bubble-Wall Plot is a one-dimensional tool not provided. Line Charts have the worst user preference for *distribution* among current visualization approaches. There is a different viewpoint on Line Charts for *distribution*: one study states that Line Charts have better accuracy and better response time for *distribution*. In comparison, another study contends they have worse accuracy and slower response time. Scatter Plots have the best response times and worse user preferences for distribution but have better accuracy than Line Charts. No previous studies have reviewed this feature in Glyphs-based approaches, Parallel Coordinate Plots, Pixel-based techniques, Radial visualizations, and TreeMaps.

- Others

The features for the other categories focus on four subcategories: *Performance Speed, Hierarchical Data, Data/Ink Ratio,* and *Difficulty in Ratios,* including:

- *Performance Speed* depends on the monitoring system’s data output. The Bubble-Wall Plot has been applied to a gas warning system (see Chapter 5—Case Study). The Line Chart is surprising in its overall low performance compared with current visualization approaches. No previous studies have discussed this feature in Glyphs-based approaches, Parallel Coordinate Plots, Pixel-based techniques, Radial visualizations, Scatter Plots, or tree maps.
- For *Hierarchical Data*, the Bubble-Wall Plot is designed with a one-dimensional approach and does not have hierarchical data issues. A TreeMap can only be applied to *hierarchical data*. No previous studies have reported this feature in Glyphs-based approaches, Line Charts, Parallel Coordinate Plots, Pixel-based techniques, Radial visualizations, or Scatter Plots.
- For the *Data/Ink Ratio*, the Bubble-Wall Plot is a one-dimensional design that presents the data to readers with a better data/ink ratio. Among the current visualization approaches, a TreeMap has a high *data-to-ink ratio* with all levels of data details in the hierarchy. No previous studies have argued for this feature in Glyphs-based approaches, Line Charts, Parallel Coordinate Plots, Pixel-based techniques, Radial visualizations, and Scatter Plots.
- For *Difficulty in Ratios*, the Bubble-Wall Plot has no difficulty in ratios. Among current visualization approaches, Line Charts are limited in terms of ratios. TreeMaps are limited to extreme aspect *ratios.* No previous studies have reported this feature in Glyphs-based approaches, Parallel Coordinate Plots, Pixel-based techniques, Radial visualizations, or Scatter Plots.

Overall, the above comparative analysis demonstrates the superiority of this new DAP visualization tool against existing data visualization tools based on five categories (Style, Value, Change, Correlation, and Others) and 26 subcategories of metric features.

### 4.6 Remarkable characteristics

**Fig 4:**
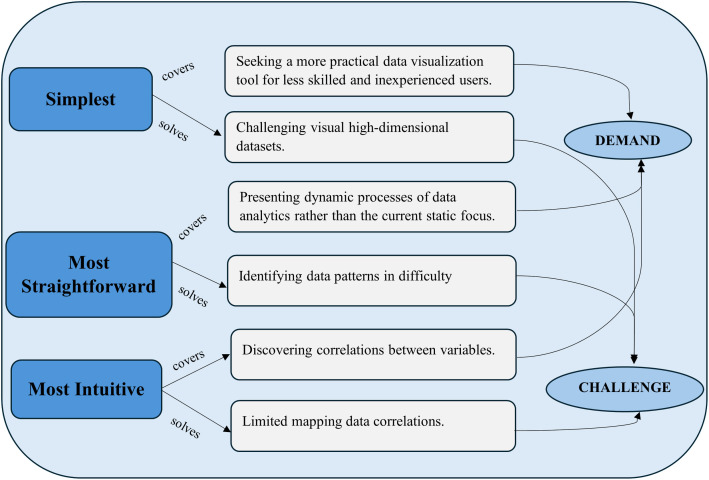
Three Remarkable Characteristics Covering Three Demands and Solving Three Challenges.

The proposed DAP visualization tool appears to have three remarkable characteristics compared with other approaches: the simplest cartographic tool, the most straightforward vision tool, and the most intuitive tool. Fig 4 illustrates that these remarkable characteristics cover three demands: seeking more practical visualization tools for less skilled or inexperienced users, presenting dynamic analytical processes of hazard data rather than current static dataset focus, and discovering correlations between various dataset variables (see section 2.1 Demands). They also address three challenges faced by existing data visualization approaches: challenging high-dimensional datasets, difficulties in identifying data patterns, and limited mapping data correlations (see section 2.2 Challenges).

The first significant characteristic is that the Bubble-Wall Plot for a dynamic analytical process is the simplest cartographic symbolization, comprising one bubble and two lines with one dimension. Among existing visualization approaches, Line Charts and Scatter Plots have two dimensions for symbolization. Glyphs-based approaches, Parallel Coordinate Plots, Pixel-based techniques, Radial visualizations, and TreeMaps use multi-dimensions. Compared with existing visualization tools, less skilled and less experienced users can easily understand and handily use this new tool without any statistical background or experience. This significant characteristic covers the primary demand (seeking a more practical data visualization tool for less skilled (Section 2-Demand) and inexperienced users). It also solves the first challenge (challenging visual high-dimensional datasets) (Section 2-Challenges).

The second impressive characteristic is that the Bubble-Wall Plot for a dynamic analytical process has the most straightforward visual effection to data analytics processes. This Bubble-Wall Plot enables quick visualization of changes in the data analytics process. The circle shape is adopted to develop this tool with three pure color hues: blue, yellow, and red. The blue circle indicates the normal status. The safety-responsive management team should not take action. The sharp yellow circle indicates the anomaly status. Although this does not state any risks, a safety-responsive management team should check the monitoring system to identify potential hazards. The sharp red circle indicates the warning status. The safety-responsive management team must immediately take action in case of a warning status. Thus, this impressive characteristic covers the second demand (presenting dynamic analytical processes of hazard data rather than the current static dataset focus.) and solves the second challenge (difficulties in identifying data patterns).

Another notable characteristic is that the Bubble-Wall Plot for dynamic data visualization is the most intuitive tool for illustrating correlations between dataset variables. This novel DAP visualization tool presents correlations between dataset variables from various inputs (see Appendix 4). This notable characteristic covers the third demand (discovering correlations between dataset variables) and solves the third challenge (limited mapping data correlations).

## 5. Case study

Robust evidence is crucial for proving the appropriate design of data visualization tools [65]. To verify the effectiveness and superiority of the DAP visualization tool, a case study using real-world datasets is conducted to demonstrate how this tool can be effectively applied to develop a gas warning system for presenting dynamic analytical processes of gas hazards.

### 5.1 Problem definition and case study background

As the world’s largest coal producer, China’s coal mining industry accounted for approximately 46% of global coal production in 2020 [66,67]. Although China has continuously increased investments in coal mine safety, its accident mortality rate is still higher than the world average: the number of significant accidents is not significantly lower than five years ago [68]. Among the present coal mining risks, methane gas (called gas in this paper) explosions or ignition in underground mines remain ever-present [69]. Existing gas monitoring systems primarily detect real-time data obtained from gas sensors. If the gas data readings reach or exceed the threshold limit value (TLV), the gas monitoring system will notify the mine’s safety-responsive team [54,70]. Previous research has identified that the current coal mining industry is seeking a new approach to exploring innovative warning systems to identify risks and improve coal mining safety [71–73].

**Fig 5:**
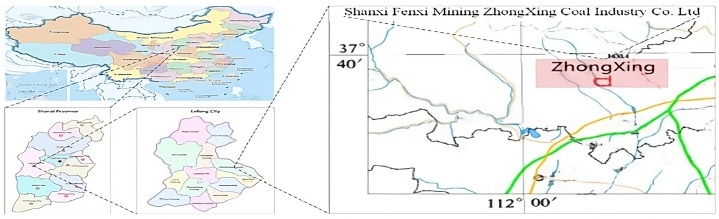
Location of the Case Study Mine -Republished from [54] under a CC-BY 4.0 license, with permission from AIS, original copyright 2021.

In this research, the ZhongXing Coal Industry Co. Ltd. (ZhongXing) was chosen as the case study mining site (Fig 5). Zhongxing is a large coal mining company owned by Shanxi Coking Coal Group Co. Ltd, China’s largest coking coal supplier, which was ranked 485th in the 2020 Fortune Global 500 (SXCC 2020).

### 5.2 Use the Bubble-Wall Plot for this case study

This subsection demonstrates how to use the Bubble-Wall Plot to develop a gas warning system that presents dynamic analytical processes of gas hazards. The procedure consists of the following seven steps:

- Step 1. Data collection

Data were collected from the gas sensor (T050204) and temperature sensor (WD050301) of the gas monitoring system in Case Study mining site No.1217 between 00:00:00 am and 23:59:59 PM on December 5, 2021 (see Appendix 4). Data were collected at 15-s intervals. The data supporting this study are available in the public domain: Zenodo (license CC BY4.0 from https://doi.org/10.5281/zenodo.7089723).

- Step 2. Define the target variable

A gas monitoring system has demonstrated an implicit correlation between gas and temperature data [53]. Thus, the target variable for this problem is chosen to be the correlation coefficient denoted as CAV, which can be calculated by equation (1) as:


$$\begin{array}{c} CAV=T*Weights\;of\;T+WD*Weights\;of\;WD \end{array}$$ (1)

Where T and WD denote the gas and temperature, the weight of T and WD denote the weight of gas and temperature, respectively. We can calculate these two weights using [54] methods as the weight of T = 0.7545 and WD = 0.2455.

- Step 3. Define the sections of CAV values

The CAV values can be split into one normal segment, one low anomaly segment, and two high anomaly sections, which are classified as high abnormal and high warning.

- Step 4. Build the Wall

ULV and LLV determine the Wall. In this case, based on the data analysis, ULV = 6.4247 and LLV = 5.0634 can be calculated.

- Step 5. Determine the TLV

In this case, the high TLV is defined by a regulatory body in the Chinese coal mining industry as 0.8 units.

Table 1 shows the data recorded between 14:43 and 15:27. At 15:27, CAV (6.4300) exceeded the ULV (6.4247).

**Table 1:** Data Analysis.

Date	Time	T	Weight of T	WD	Weight of WD	ULV	LLV	CAV	Exceeding the ULV
2021-12-05	14:43	0.18	0.7545	24.8	0.2455	**6.4247**	5.0634	6.2242	no
2021-12-05	14:50	0.19		24.8				6.2318	no
2021-12-05	14:52	0.20		24.8				6.2393	no
2021-12-05	14:55	0.21		24.8				6.2469	no
2021-12-05	14:56	0.22		24.8				6.2544	no
2021-12-05	14:57	0.23		24.8				6.2620	no
2021-12-05	15:02	0.24		24.8				6.2695	no
2021-12-05	15:05	0.25		24.8				6.2770	no
2021-12-05	15:10	0.26		25.3				6.4073	no
2021-12-05	15:12	0.27		25.3				6.4149	no
2021-12-05	15:15	0.28		25.3				6.4224	no
2021-12-05	15:27	0.29		25.3				6.4300	yes

(Note: Cav = T*weights of T + WD*weights of WD)

- Steps.6-7. Implement and use of the Bubble-Wall Plots

### 5.3 Performance benchmarks

The Bubble-Wall Plot is performed at an interval of 15 s based on data collected from the monitoring system in the Case Study Mine. The tool’s effectiveness is approximately synchronous with the monitoring system’s data feed. For more details about data processing in the case study mine, see our previous study [54].

The tool can handle both real-time and static data simultaneously. If the data fed by the monitoring systems is real-time datasets, the tool will produce a dynamic visualization system. If static datasets, such as historical datasets, are used to provide data, the tool will produce static visualization outputs.

## 6. Conclusions, Recommendations, Implication, and Further Studies

### 6.1 Conclusions

This paper presents the DAP visualization tool based on the Bubble-Wall Plot and the procedure of using this tool for a given use case. Comparative analysis is used to identify the need for a new anomaly data visualization tool, raise awareness of different views and opinions on how to use existing data visualization tools efficiently, and also convince the superiority of this new tool based on five categories and 26 subcategories.

This new DAP visualization tool has three remarkable characteristics compared with other approaches: the simplest cartographic tool, the most straightforward visual tool, and the most intuitive tool. It is practical to meet the three identified demands and address the challenges faced by the existing data visualization approaches.

This new tool is applicable to any use case or problem that has a dynamic analytic process to support decision marking through monitoring a target variable. A case study of a gas early warning system in a coal mining industry demonstrates the effectiveness of this new tool. More importantly, this DAP visualization tool can be used to develop visual warning systems that visualize dynamic analytical processes of hazard data.

### 6.2 Recommendations, Implications, Limitations, and Further Studies

Based on the above findings and discussions, this research provides two recommendations, one implication, six limitations, and eleven further study topics.

- **Recommendations**

The primary recommendation is that Appendix 3 can guide researchers and practitioners to select the most suitable data visualization tool for analyzing hazard data. They can use metric features from five categories (Style, Value, Change, Correlation, and Others) and 26 subcategories to match their business or industry needs.

Another recommendation is to introduce data visualization courses into information systems (IS) and business programs in higher education. Most domain practitioners are not yet familiar with the advantages of interactive visualization [5]. Many scientists and researchers are rarely trained to develop practical visualization tools [2].

- **Implications and further studies**

The remarkable characteristics of the Bubble-Wall Plot for dynamic analytical processes imply that the proposed tool can be used in other industrial applications and hazard warning systems. However, the critical challenge associated with using this tool is identifying the strong correlations between datasets fed by monitoring systems. The approach has been well-discussed in our previous studies on how to conduct correlation analysis to prove whether a strong relationship or evidence exists between various datasets, such as [54,70,74,75]. The following further study (FS) should be conducted:

- FS1: Developing a guide for generalizing to other domains requiring different types of data visualization, which can be used to demonstrate how researchers and practitioners may use this tool effectively to visualize dynamic analytical processes for hazard data in developing hazard-warning systems in broader applications, such as finance, healthcare, environmental systems, etc.,

- Limitations and further studies

The main limitation of this research is that it has applied four elements of Bertin’s cartographic symbolization for the Bubble-Wall Plot, including color, shape, position, and orientation. Still, it does not apply the aspects of texture, color value (lightness or darkness), and size. Symbol size carries a highly effective message: increasing icon size indicates increased risks [58]. The following FSs should be explored:

- FS2: Adding texture and color value (lightness or darkness) to design new DAP tools further.
- FS3: Exploring how the size feature can be added to develop an updated Bubble-Wall Plot to fit more business applications in various industries.

The second limitation is that this research finds varying viewpoints compared to previous studies on visualization features, but in-depth research is needed in this project. The following FSs are valuable for conducting further investigations, including:

- FS4: Different views of Line Charts.
- FS5: Different perspectives on Scatter Plots.
- FS6: Arguments between Scatter Plots and Line Charts.
- FS7: Diverse opinions between Parallel Coordinate Plots and Scatter Plots.

The third limitation is that this research focuses on dynamic data; however, it must explore how well the tool handles static or hybrid data (static and streaming). This limitation may reduce flexibility in industrial applications requiring both data types. The following FS is valuable to be conducted as:

- FS8: Exploring how well this tool can be adapted to handle hybrid data mixed with static and real-time streaming.

The fourth limitation is that this research proposes a DAP visualization tool based on the Bubble-Wall Plot- and presents a case study to demonstrate how this tool can be effectively applied to develop a gas warning system. The dynamic analytical processes of gas hazards are also presented. However, this study needs to conduct a user evaluation of the tool’s effectiveness due to the project’s restriction on limited users who can operate the warning system. The following FS is required:

- FS9: Conducting user evaluations incorporating user-centric feedback to enhance understanding of the tool’s practical utility.

The fifth limitation is that this research does not discuss whether this tool may be integrated with machine learning (ML) algorithms to develop more effective early warning systems. Recent research highlights that using efficient ML algorithms with better performance for short-term forecasting may reduce the risk of accidents such as gas explosions, safeguard workers, and enhance the ability to prevent and mitigate disasters [75]. ML is used in graphs for data visualization purposes to be ubiquitous [76]. There is a need to explore the potential of integrating ML with the development of visualization tools [25] Our recent study highlights that although no single algorithm can fit all applications, three optimal algorithms - Linear Regression (LR), Random Forest (RF), and Support Vector Machine (SVM) – are more efficient ML algorithms with better performance than the other short-term forecasting algorithms. The following FS needs to be addressed as:

- FS10: Exploring a better approach to integrating a Bubble-Wall Plot with an optimal ML algorithm to develop more effective early-warning systems to fit industry needs in hazard prediction.

The sixth limitation is that this research does not discuss considerations of interactive visualization tools impacting ethics and does not consider whether any temporal biases could impact political effects when presenting data visualizations. There is a need to discuss the added value of interactive visualization for bioethics and must also consider some of the limitations that shadow developing visualization tools using new technologies and innovations, such as accessibility, web content, digital divide, misuse of aesthetic appeal, trust, and confidence [77]. Temporal bias in visualization and related political effects must also be explored [78]. The impact of temporal biases and political effects on data visualizations needs to be discussed in further research:

- FS11: Exploring ethics considerations and the political effects of temporal bias in data visualization tools.

## Supporting information

S1 File Appendices. (PDF)
