# Supplementary material for: Bubble-Wall Plot as a Dynamic Analytical Processing Visualization Tool for Developing Visual Warning Systems: a Case Study
Source: PLoS One. 2025 Jul 1;20(7):e0321077. doi: 10.1371/journal.pone.0321077 (PMC12212487; doi:10.1371/journal.pone.0321077)
Supplement: S1 File — (PDF) [file pone.0321077.s001.pdf]

Appendix 1: Data Visualisation Tools in Q1 Publications Between 2017 and 2024

Approach	Legend	Reference	Approach	Legend	Reference
Bar Chart	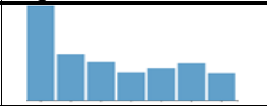	[1-9]	Radial	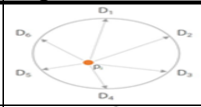	[10-12]
Bubble Chart	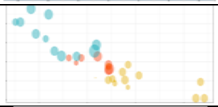	[13, 14]	Radar Chart	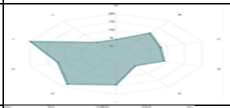	[13, 15]
Dimension Hierarchies	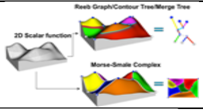	[10]	Sankey Chart	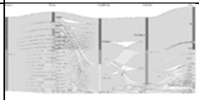	[2, 16]
Geo-Spatial	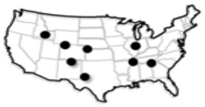	[7, 12]	Scatter Plot	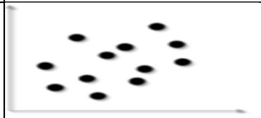	[1, 3-5, 9, 10, 12, 13, 15, 17-22]
Glyphs	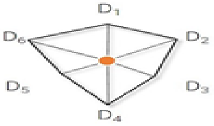	[10, 12, 16]	Stacked Graph	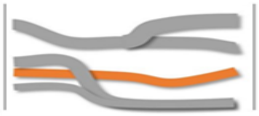	[2, 9, 12, 15]
Heatmap	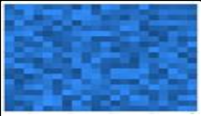	[4, 5, 13, 16, 23-25]	Sunburst	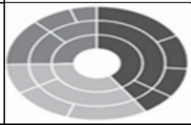	[2, 16]
Line Chart	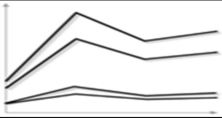	[1-3, 5, 9, 12, 25]	Table Lens	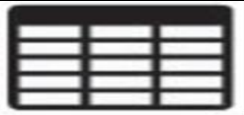	[1, 9, 10]
Network Diagrams	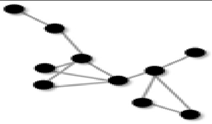	[7, 12, 17]	TreeMap	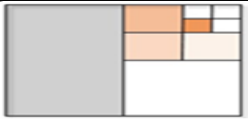	[2, 5, 12, 13, 16, 24, 26]
Parallel Coordinates	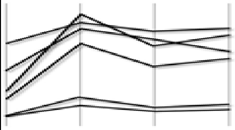	[2, 9, 10, 12, 15, 16, 18, 21, 27]	Typographic	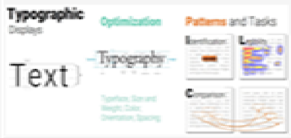	[12]
Pie Chart	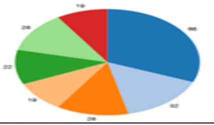	[1-3, 7, 9, 14]	Tag Clouds	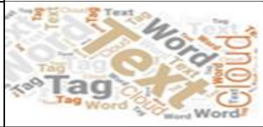	[2, 6, 12]
Pixel	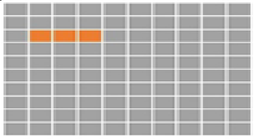	[10, 12]	Weather Map	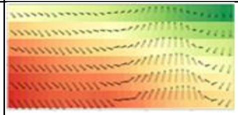	[13]
Polar Coordinate	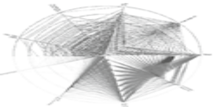	[10, 11, 16]	Violin Plot	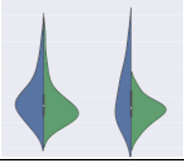	[4]

Appendix 2: Reviews of Metric Features of Existing Data Visualisation Tools

Features	Accuracy				Time				User Preference				reference
	Best	Better	Worse	Worst	Best	Better	Worse	Worst	Best	Better	Wo rse	Worst	
Find Anomalies	SP	LC PCP Pixel TreeMap Glyph Radial				SP PCP Pixel TreeMap Glyph Radial	LC		SP	PCP Pixel TreeMap Glyph Radial	LC		[9, 12, 15, 28]
Correlation	LC	SP PCP Pixel Radial			LC	SP PCP Pixel Radial			LC	PCP Pixel Radial	SP		[9, 15, 18, 21, 29]
Distribution		SP>LC	LC(d)		SP(d)	LC SP	LC(d)				SP	LCl	[3, 9]
Ordering of Dimension		SP	LC	PCP Pixel Radial		LC >SP		PCP PixelRadial			LC >SP	PCP Pixel Radial	[9, 12, 30]
Filtering		LC>SP PCP				SP PCP	LC>SP(d)			PCP	LC >SP		[3, 9, 15, 18, 27]
Find Clusters /Clustering		GlyphsPixel SP> PCP TreeMap	LC SP(d)			SP >PCP Pixel TreeMap Glyph	LC			Pixel TreeMap Glyph	LC >SP		[3, 9, 12, 18, 31]
Retrieve Value		SP	LC	TreeMap Glyph Radial			LC>SP	TreeMap Glyph Radial			LC >SP	TreeMap Glyph Radial	[9, 11, 12, 26]
Find Extremum	SP	LC PCP		TreeMap Glyph		PCP LC>SP		TreeMap Glyph		PCP	LC >SP	TreeMap Glyph	[9, 12, 26]
Derived Value		SP	LC	SP(d)>PCP TreeMap Glyph Radial			LC SP	TreeMap SP(d)> PCPGlyph Radial			SP	LC TreeMap SP(d)>PCP Glyph Radial	[9, 11, 12, 18, 26, 28]
Determine Range		SP>LC			SP	LC			SP	LC			[9]
Low Dimension	PCP > SP; in accuracy,in time SP > PCP(d)												[11, 12, 18, 32]

	Pixel Radial												[9]
**High Dimension**	SP > PCP; PCP > SP(d); Treemap; Glyphs												[11, 12, 18, 32]
	Pixel Radial												
**Interaction**	not recommend for Radial; SP												[11, 20]
**Layout Flexibility**	Glyph TreeMap												[12, 33]
**Overview of Data**	PCP SP												[15, 20, 27]
**Overplotted**	Pixel (no);				PCP (yes) Radial(yes); SP(yes)								[11, 12, 19]
**Change Detection**		PCP SP				PCP SP				SP			[28]
**Perceived Signal**						SP	PCP						[34]
**Judgment**		SP	LC	PCP TreeMap									[12, 34, 35]
**Performance Speed**	LC												[9]
**Hierarchical Only**	TreeMap												[36]
**Data/Ink Ratio**	TreeMap												[37]
**Difficult to Ratios**	TreeMap												[12, 35]
	LC												
**Details Limited**	Radial SP												[20, 38]

(Note: LC stands for Line Chart; PCP stands for Parallel Coordinates Plot; SP stands for Scatter Plot; > stands for being better; (d) stands for different opions by the research)

Deep blue color stands for best; blue stands for better; Light orange stands for worse; Dark orange stands for worst

Appendix 3: Feature Comparison Between Bubble-Wall Plot and Other Anomaly Visualization Tool

Approaches Features		Bubble-Wall	Glyphs	Line Chart	Parallel Coordinates	Pixel	Radial	Satter Plot	TreeMap
Style	**Symbolization Outlier***	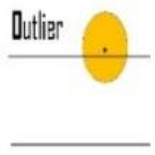	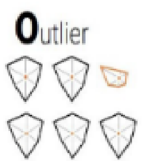	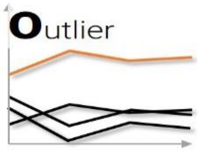	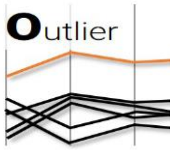	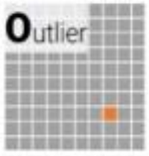	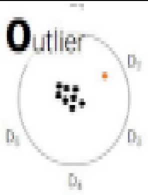	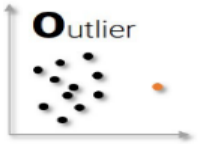	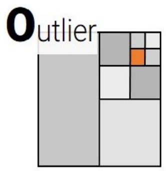
	**Style**	simplest	complex	simple	complex	complex	complex	simple	complex
	**Dimension**	one	multi	two	multi	multi	multi	two-dimensional and be extensible to multi-dimensional data	multi
	**Ordering of Dimensions**	yes		worse in accuracy; better in time; worse in preference	worst		worst	better in accuracy; better in time; worse in preference	
	**Low Dimensions**	yes			much better; better(d)	best	yes	better; much better (d)	
	**High Dimensions**	yes	better		better, much better(d)	better	yes	much better; better (d)	better
	**Interaction**	yes					no	no	
	**Layout Flexibility**	yes	best						best
Value	**Find Anomalies**	simplest	yes	better in accuracy; worse in time; worse in preference	yes	yes	yes	best in accuracy, better in time, better in preference	yes
	**Find Extremum**	yes	worst	better in accuracy; better in time, worse in preference; worse in time	better	better in accuracy		best in accuracy, better in time, worse in preference	worst
	**Retrieve Value**	yes	worst	worst	worst		worst	better, worst(d)	worst
	**Derived Value**	yes	worst	worse			worst	better in accuracy, worse in time, worse in preference	worst

	**Details Limited**	no					yes	yes	
	**Data Overview**	yes			best			best	
	**Overplotted**	no			yes	no	yes	yes	
**Change**	**Determine Range**	yes		better				better	
	**Change Detection**	yes			better in accuracy, better in time				
	**Perceived Signal**	yes			worse in time			better in time	
	**Judgment**	yes		worse in accuracy	worst in accuracy			better in accuracy	worst in accuracy
**Correlation**	**Correlation**	indirect		best	better			better in accuracy, better in time, better in preference; worse in preference(d)	
	**Find Clusters**	no		worse	better in accuracy, better in time	better		better in accuracy, better in time; worse in preference; worse in accuracy(d)	better
	**Filtering**	no		better in accuracy worse in time, worse in preference	bettter			better in accuracy; better in time worse in preference; worse in time(d)	
	**Characterize Distribution**	no		better in accuracy, better in time, worst(d)				better in accuracy; better in time, worse in preference	
**Others**	**Performance Speed**	yes		worst					
	**Hierarchical Data Only**	no							best
	**Data/Ink Ratio**	yes							best
	**Difficult in Ratios**	no		yes					yes

(Note: * stands for reviewed by Behrisch et al. (2018, pp.635-647) except for Bubble-Wall Plot; Time stands for response time; Blank stands for no reported literature)

Appendix 4: A Three-Layer Demo for Illustrating The Use of Bubble-Wall Plot

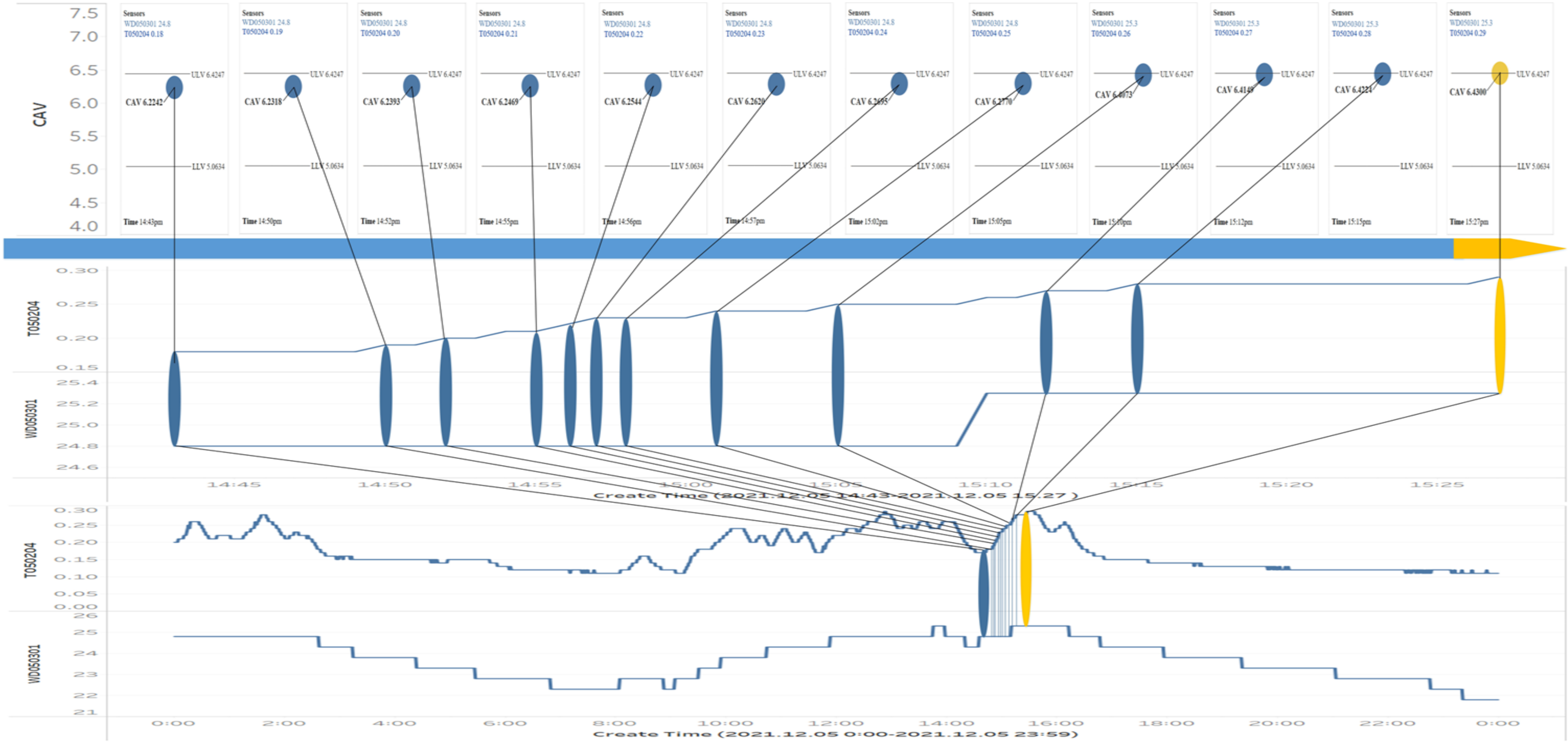

References

1. Saket B, Endert A, Demiralp Ç. Data and Task Based Effectiveness of Basic Visualizations. IEEE Transactions on Visualization and Computer Graphics. 2017;PP. doi: 10.1109/TVCG.2018.2829750.
2. Perkhofer LM, Hofer P, Walchshofer C, Plank T, Jetter HC. Interactive visualization of big data in the field of accounting: A survey of current practice and potential barriers for adoption. Journal of applied accounting research. 2019;20(4):497-525. doi: 10.1108/JAAR-10-2017-0114.
3. Zhang D, Sarvghad A, Miklau G. Investigating Visual Analysis of Differentially Private Data. IEEE transactions on visualization and computer graphics. 2021;27(2):1786-96. doi: 10.1109/TVCG.2020.3030369.
4. Joshi J, Blankenberg D. PDAUG: a Galaxy based toolset for peptide library analysis, visualization, and machine learning modeling. BMC Bioinformatics. 2022;23(1):197. Epub 20220528. doi: 10.1186/s12859-022-04727-6. PubMed PMID: 35643441; PubMed Central PMCID: PMCPMC9148462.
5. Mishra P, Kumar S, Chaube MK. Graph Interpretation, Summarization and Visualization Techniques: A Review and Open Research Issues. Multimedia Tools and Applications. 2022;82(6):8729-71. doi: 10.1007/s11042-021-11582-9.
6. Mollayeva T, Tran A, Chan V, Colantonio A, Escobar MD. Sex-specific analysis of traumatic brain injury events: applying computational and data visualization techniques to inform prevention and management. BMC Medical Research Methodology. 2022;22(1):30. Epub 20220130. doi: 10.1186/s12874-021-01493-6. PubMed PMID: 35094688; PubMed Central PMCID: PMCPMC8802441.
7. Cobilean V, Mavikumbure HS, McBride BJ, Vaagensmith B, Singh VK, Li R, et al. A Review of Visualization Methods for Cyber-Physical Security: Smart Grid Case Study. IEEE Access. 2023;11:59788-803. doi: 10.1109/access.2023.3286304.
8. Samadbeik M, Engstrom T, Lobo EH, Kostner K, Austin JA, Pole JD, Sullivan C. Healthcare dashboard technologies and data visualization for lipid management: A scoping review. BMC medical informatics and decision making. 2024;24(1):352-16. doi: 10.1186/s12911-024-02730-w.
9. Saket B, Endert A, Demiralp C. Task-Based Effectiveness of Basic Visualizations. IEEE Transactions on Visualization and Computer Graphics. 2019;25(7):2505-12. Epub 20180504. doi: 10.1109/TVCG.2018.2829750. PubMed PMID: 29994001.
10. Shusen L, Maljovec D, Bei W, Bremer P-T, Pascucci V. Visualizing High-Dimensional Data: Advances in the Past Decade. IEEE transactions on visualization and computer graphics. 2017;23(3):1249-68. doi: 10.1109/TVCG.2016.2640960.
11. Albo Y, Lanir J, Bak P, Rafaeli S. Off the Radar: Comparative Evaluation of Radial Visualization Solutions for Composite Indicators. IEEE transactions on visualization and computer graphics. 2016;22(1):569-78. doi: 10.1109/TVCG.2015.2467322.
12. Behrisch M, Blumenschein M, Kim NW, Shao L, El‐Assady M, Fuchs J, et al. Quality Metrics for Information Visualization. Computer graphics forum. 2018;37(3):625-62. doi: 10.1111/cgf.13446.
13. Cui L, Liu Z. Synergy between research on ensemble perception, data visualization, and statistics education: A tutorial review. Attention, perception & psychophysics. 2021;83(3):1290-311. doi: 10.3758/s13414-020-02212-x.
14. Samadbeik M, Engstrom T, Lobo EH, Kostner K, Austin JA, Pole JD, Sullivan C. Healthcare dashboard technologies and data visualization for lipid management: A scoping review. BMC Medical Informatics and Decision Making. 2024;24(1):352. Epub 20241121. doi: 10.1186/s12911-024-02730-w. PubMed PMID: 39574106; PubMed Central PMCID: PMCPMC11583543.

15. Johansson J, Forsell C. Evaluation of Parallel Coordinates: Overview, Categorization and Guidelines for Future Research. IEEE transactions on visualization and computer graphics. 2016;22(1):579-88. doi: 10.1109/TVCG.2015.2466992.
16. Perkhofer L, Walchshofer C, Hofer P. Does design matter when visualizing Big Data? An empirical study to investigate the effect of visualization type and interaction use. Journal of management control. 2020;31(1-2):55-95. doi: 10.1007/s00187-020-00294-0.
17. Bendoly E. Fit, Bias, and Enacted Sensemaking in Data Visualization: Frameworks for Continuous Development in Operations and Supply Chain Management Analytics. Journal of business logistics. 2016;37(1):6-17. doi: 10.1111/jbl.12113.
18. Netzel R, Vuong J, Engelke U, O'Donoghue S, Weiskopf D, Heinrich J. Comparative eye-tracking evaluation of scatterplots and parallel coordinates. Visual informatics (Online). 2017;1(2):118-31. doi: 10.1016/j.visinf.2017.11.001.
19. Urribarri DK, Castro SM. Prediction of data visibility in two-dimensional scatterplots. Information visualization. 2017;16(2):113-25. doi: 10.1177/1473871616638892.
20. Nguyen QV, Miller N, Arness D, Huang W, Huang ML, Simoff S. Evaluation on interactive visualization data with scatterplots. Visual informatics (Online). 2020;4(4):1-10. doi: 10.1016/j.visinf.2020.09.004.
21. Abi Akle A, Yannou B, Minel S. Information visualisation for efficient knowledge discovery and informed decision in design by shopping. Journal of engineering design. 2019;30(6):227-53. doi: 10.1080/09544828.2019.1623383.
22. Blanchard AM, Taylor A, Warry A, Shephard F, Curwen A, Leigh JA, et al. PIMMS-Dash: Accessible analysis, interrogation, and visualisation of high-throughput transposon insertion sequencing (TIS) data. Computational and Structural Biotechnology Journal. 2024;23:3780-3. Epub 20241018. doi: 10.1016/j.csbj.2024.10.025. PubMed PMID: 39525080; PubMed Central PMCID: PMCPMC11550763.
23. Barter RL, Yu B. Superheat: An R Package for Creating Beautiful and Extendable Heatmaps for Visualizing Compi-x Data. Journal of computational and graphical statistics. 2018;27(4):910-22. doi: 10.1080/10618600.2018.1473780.
24. Frantz TL. Blockmap: an interactive visualization tool for big-data networks. Computational and mathematical organization theory. 2018;24(2):149-68. doi: 10.1007/s10588-017-9252-6.
25. Sleigh J, Ormond K, Schneider M, Stern E, Vayena E. How Interactive Visualizations Compare to Ethical Frameworks as Stand-Alone Ethics Learning Tools for Health Researchers and Professionals. AJOB Empirical Bioethics. 2023;14(4):197-207. Epub 20230419. doi: 10.1080/23294515.2023.2201479. PubMed PMID: 37074681.
26. Cheong L, Bleisch S, Kealy A, Tolhurst K, Wilkening T, Duckham M. Evaluating the impact of visualization of wildfire hazard upon decision-making under uncertainty. International journal of geographical information science : IJGIS. 2016;30(7):1377-404. doi: 10.1080/13658816.2015.1131829.
27. Zhang C, Gong Q-w, Koyamada K. Visual analytics and prediction system based on deep belief networks for icing monitoring data of overhead power transmission lines. Journal of visualization. 2020;23(6):1087-100. doi: 10.1007/s12650-020-00670-x.
28. Kanjanabose R, Abdul-Rahman A, Chen M. A Multi-task Comparative Study on Scatter Plots and Parallel Coordinates Plots. Computer graphics forum. 2015;34(3):261-70. doi: 10.1111/cgf.12638.
29. Harrison L, Fumeng Y, Franconeri S, Chang R. Ranking Visualizations of Correlation Using Weber's Law. IEEE transactions on visualization and computer graphics. 2014;20(12):1943-52. doi: 10.1109/TVCG.2014.2346979.

30. Bertini E, Tatu A, Keim D. Quality Metrics in High-Dimensional Data Visualization: An Overview and Systematization. IEEE transactions on visualization and computer graphics. 2011;17(12):2203-12. doi: 10.1109/TVCG.2011.229.
31. Soares AGM, Miranda ETC, do Amor Divino Lima RS, dos Santos CGR, Meiguins BS. Depicting more information in enriched squarified treemaps with layered glyphs. Information (Basel). 2020;11(2):123. doi: 10.3390/info11020123.
32. Kuang X, Zhang H, Zhao S, McGuffin MJ. Tracing Tuples Across Dimensions: A Comparison of Scatterplots and Parallel Coordinate Plots. Computer graphics forum. 2012;31(3pt4):1365-74. doi: 10.1111/j.1467-8659.2012.03129.x.
33. Kopp W, Weinkauf T. Temporal Treemaps: Static Visualization of Evolving Trees. IEEE transactions on visualization and computer graphics. 2019;25(1):534-43. doi: 10.1109/TVCG.2018.2865265.
34. Li J, Martens J-B, van Wijk JJ. Judging Correlation from Scatterplots and Parallel Coordinate Plots. Information visualization. 2010;9(1):13-30. doi: 10.1057/ivs.2008.13.
35. Heer J, Agrawala M. Multi-Scale Banking to 45 Degrees. IEEE transactions on visualization and computer graphics. 2006;12(5):701-8. doi: 10.1109/TVCG.2006.163.
36. Wang L, Wang G, Alexander CA. Big Data and Visualization: Methods, Challenges and Technology Progress. Digital Technologies. 2015;1(1):33-8. doi: 10.12691/dt-1-1-7.
37. Songer AD, Hays B, North C. Multidimensional visualization of project control data. Construction innovation. 2004;4(3):173-90. doi: 10.1108/14714170410815088.
38. Keim DA, Mansmann F, Schneidewind J, Schreck T, editors. Monitoring Network Traffic with Radial Traffic Analyzer2006: IEEE.
